# Supplementary material for: Association Study between Antioxidant Nutrient Intake and Low Bone Mineral Density with Oxidative Stress-Single Nucleotide Variants: GPX1 (rs1050450 and rs17650792), SOD2 (rs4880) and CAT (rs769217) in Mexican Women
Source: Antioxidants (Basel). 2023 Dec 8;12(12):2089. doi: 10.3390/antiox12122089 (PMC10740963; doi:10.3390/antiox12122089)
Supplement: Supplementary file 1 [file antioxidants-12-02089-s001.zip › antioxidants-2723952-supplementary.docx]

| **Supplementary Table S1.** Demographic characteristics of the study population. | | | | |
| --- | --- | --- | --- | --- |
|  | **Total** | **<45 years** | **≥45 years** |  |
| **Variable** | n=1269 | n=377 | n=892 | *p* value |
| Age (years) | 53(42-62) | 34(26-41) | 58(52-65) | <0.001 |
| BMI^a^ (kg/m^2^) | 26.7(23.9-30.0) | 25.0(22.6-28.2) | 27.4(24.5-30.6) | <0.001 |
| Overweight (%) | 40.1 | 32.4 | 43.3 | <0.001 |
| Obesity (%) | 25.2 | 18.0 | 28.3 | <0.001 |
| Glucose (mg/dL) | 95(90-104) | 91(87-96) | 98(92-108) | <0.001 |
| IFG^b^ (%) | 5.3 | 1.3 | 7.0 | <0.001 |
| Diabetes (%) | 16.6 | 4.5 | 21.6 | <0.001 |
| Triglycerides (mg/dL) | 148(107-198) | 127(90-174) | 158(115-208) | <0.001 |
| LDL (mg/dL) | 121(99-145.9) | 106.3(92-129.9) | 127.1(105-151) | <0.001 |
| Total cholesterol (mg/dL) | 198(172-226) | 181(159-205) | 206(179-236) | <0.001 |
| High cholesterol (%) | 48.7 | 30.8 | 56.3 | <0.001 |
| Energy (kcal/día) | 1701(1283-2233) | 1786(1333-2375) | 1684(1269-2137) | 0.0182 |
| BMD^c^ total hip (g/cm^2^) | 0.968(0.873-1.069) | 1.028(0.944-1.125) | 0.940(0.851-1.037) | <0.001 |
| Low BMD^c^ total hip (%) | 25.2 | 9.3 | 32.0 | <0.001 |
| BMD^c^ femoral neck (g/cm^2^) | 0.934(0.824-1.028) | 1.018(0.937-1.105) | 0.894(0.805-0.985) | <0.001 |
| Low BMD^c^ femoral neck (%) | 38.6 | 14.1 | 49.0 | <0.001 |
| BMD^c^ lumbar spine (g/cm^2^) | 1.070(0.956-1.175) | 1.168(1.088-1.264) | 1.014(0.915-1.129) | <0.001 |
| Low BMD^c^ lumbar spine (%) | 49.3 | 19.4 | 62.0 | <0.001 |
| Folate (µg/day) | 292.5(211.3-409.3) | 292.5(213.2-414.6) | 288.9(210-406.7) | 0.9903 |
| Retinol (UI/day | 7232(6784-7813) | 7038(6602-7420) | 7326(6867-7967) | <0.001 |
| Selenium (µg/day) | 39.2(30.6-49.2) | 42.6(33.1-53.5) | 38.1(29.4-47.3) | <0.001 |
| Vitamin C (mg/day) | 308.8(231.3-388.5) | 274.4(200.9-351) | 319.3(242.3-402.3) | <0.001 |
| Vitamin E (µg/day) | 6.8(5.8-8.0) | 6.4(5.5-7.6) | 6.9(5.9-8.2) | <0.001 |
| Zinc (mg/day) | 6.5(5.8-7.5) | 6.4(5.7-7.4) | 6.6(5.8-7.5) | 0.0386 |
| 0-4^d^ (%) | 56.4 | 49.3 | 59.4 | 0.0009 |
| 5-6 ^d^ (%) | 43.6 | 50.7 | 40.6 | 0.0009 |
| Vitamin B12 (mg/day) | 2.3(1.4-3.6) | 2.3(1.4-3.5) | 2.2(1.4-3.6) | 0.9461 |
| Omega 6 (g/day) | 10.1(7.5-14.1) | 11.0(7.9-15.1) | 9.7(7.4-13.7) | 0.0007 |
| Saturated fat (g/day) | 13(9-18.6) | 14.7(9.8-22) | 12.4(8.8-17.6) | <0.001 |
| Fiber (g/day) | 24.6(18.1-33.2) | 23.5(16.8-32.7) | 25.0(18.5-33.4) | 0.0419 |
| Riboflavin (mg/day) | 1.4(1.0-2.0) | 1.4(1.01-2.07) | 1.4(1.0-2.0) | 0.5027 |
| Vitamin B6 (mg/day) | 1.5(1.1-2.1) | 1.5(1.1-2.1) | 1.5(1.1-2.1) | 0.9415 |
| Vitamin D (UI/day) | 145.4(87.1-252.9) | 149.6(82.1-246) | 144.8(89.4-256.1) | 0.4374 |
| Vitamin E (µg/day) | 6.1(4.5-8.5) | 6.1(4.5-8.6) | 6.1(4.4-8.5) | 0.8146 |
| Median (P25-P75).  ^a^BMI = Body Mass Index.  ^b^IFG = Impaired Fasting Glucose.  ^c^BMD = Bone Mineral Density.  ^d^DAQs = Dietary antioxidant quality score. | | | | |

| **Supplementary Table S2.** Allele frequencies of the SNVs derived from 1000 Genomes Project populations and the cohort from the current study | | | | | | | |
| --- | --- | --- | --- | --- | --- | --- | --- |
| **SNV** | **Gene** | **Chr.** | **MAF** | **CEU** | **MXL** | **HWCS** | ***p* value HWE** |
| **rs1050450** | *GPX1* | 3 | A | 28.3 | 18.8 | 13.5 | 0.3584 |
| **rs17650792** | *GPX1* | 3 | A | 55.1 | 30.5 | 23.5 | 0.7196 |
| **rs4880** | *SOD2* | 6 | A | 57.1 | 35.2 | 34.5 | 0.7829 |
| **rs769217** | *CAT* | 11 | A | 19.7 | 27.3 | 28.4 | 0.1304 |
| MAF: Minor Allele Frequency  CEU: Utah Residents with Northern and Western European ancestry.  MXL: Mexican Ancestry in Los Angeles.  HWCS: Health workers cohort study.  HWE: Hardy-Weinberg Equilibrium.  *GPX1*: Glutation Peroxidase 1  *SOD2*: Superoxide Dismutase 2  *CAT*: Catalase | | | | | | | |

| **Supplementary Table S3.** Demographic characteristics of the study population by rs1050450 and rs17650792 (*GPX1*). | | | | | | |
| --- | --- | --- | --- | --- | --- | --- |
|  | **rs1050450** | | | **rs17650792** | | |
|  | GG | GA+AA |  | GG | GA+AA |  |
| **Variable** | n=954 | n=312 | *p* value | n=741 | n=516 | *p* value |
| Age (yr.) | 53(42-62) | 54(42-62) | 0.7929 | 52(42-62) | 54(43-63) | 0.0926 |
| BMI^a^ (kg/m^2^) | 26.7(24.0-30.1) | 26.8(23.8-29.8) | 0.7974 | 27(24.1-30.3) | 26.5(23.7-29.5) | 0.1374 |
| Glucose (mg/dL) | 96(90-105) | 95(90-103) | 0.4202 | 96(90-105) | 95(89-103) | 0.171 |
| IFG^b^ (%) | 5.8 | 3.9 | 0.1229 | 5.8 | 4.5 | 0.3101 |
| Diabetes (%) | 16.6 | 16.4 | 0.9013 | 17 | 16.1 | 0.6734 |
| Total cholesterol (mg/dL) | 199(172-226) | 195(171-225) | 0.376 | 197(170-225) | 199(175-230) | 0.1685 |
| High cholesterol (%) | 49.9 | 44.9 | 0.2756 | 48.5 | 49 | 0.8615 |
| Energy (kcal/día) | 1697(1269-2247) | 1713(1337-2179) | 0.930 | 1701(1282-2238) | 1700(1290-2240) | 0.724 |
| BMD^c^ total hip (g/cm^2^) | 0.967(0.873-1.076) | 0.968(0.875-1.045) | 0.6193 | 0.971(0.880-1.074) | 0.957(0.859-1.058) | 0.1485 |
| BMD^c^ femoral neck (g/cm^2^) | 0.936(0.839-1.029) | 0.922(0.824-1.025) | 0.2713 | 0.940(0.843-1.028) | 0.919(0.820-1.025) | 0.0472 |
| BMD^c^ lumbar spine (g/cm^2^) | 1.071(0.958-1.175) | 1.066(0.945-1.175) | 0.8303 | 1.071(0.968-1.175) | 1.056(0.948-1.171) | 0.2407 |
| Retinol (UI/day) | 7228(6774-7820) | 7235(6809-7798) | 0.5151 | 7237(6767-7849) | 7224(6808-7777) | 0.8289 |
| Vitamin C (mg/day) | 309(230-388) | 309(233-389) | 0.7097 | 309(228-389) | 310(234-388) | 0.4779 |
| Vitamin E (µg/day) | 6.8(5.7-8.0) | 6.8(5.9-7.9) | 0.5216 | 6.9(5.6-8.0) | 6.7(5.9-7.9) | 0.9834 |
| Selenium (µg/day) | 38.9(30-49.2) | 40(31.3-49.1) | 0.2625 | 39.2(30.6-49.7) | 39.2(30.5-47.9) | 0.4461 |
| Zinc, (mg/day) | 6.5(5.8-7.4) | 6.6(5.8-7.8) | 0.1881 | 6.5(5.8-7.3) | 6.6(5.7-7.7) | 0.2042 |
| Magnesium (mg/day) | 329(293-370) | 329(293-378) | 0.2283 | 330(294-371) | 327(292-375) | 0.873 |
| 0-4^d^ (%) | 58.0 | 51.6 | 0.0478 | 57.2 | 54.7 | 0.3795 |
| 5-6^d^ (%) | 42.0 | 48.4 | 0.0478 | 42.8 | 45.4 | 0.3808 |
| Folate (µg/day) | 315(262.9-385.7) | 320.9(266-391.9) | 0.3319 | 26.9(22.2-32.5) | 26.3(22.2-31.9) | 0.3395 |
| Vitamin B12 (mg/day) | 2.4(1.7-3.3) | 2.6(1.9-3.7) | 0.041 | 2.4(1.7-3.3) | 2.5(1.8-3.6) | 0.1281 |
| Omega 6 (g/day) | 11.3(9.3-13.1) | 11.8(9.9-13.5) | 0.0224 | 11.5(9.4-13.2) | 11.4(9-5-13.3) | 0.7445 |
| Median (P25-P75).  ^a^BMI = Body Mass Index.  ^b^IFG = Impaired Fasting Glucose.  ^c^BMD = Bone Mineral Density.  ^d^DAQs = Dietary antioxidant quality score. | | | | | | |

| **Supplementary Table S4.** Association of *GPX1*, *SOD2* and *CAT* variants with BMD (g/cm^2^) in Mexican women stratified by age. | | | | | | | | | | | | | | | | | | |
| --- | --- | --- | --- | --- | --- | --- | --- | --- | --- | --- | --- | --- | --- | --- | --- | --- | --- | --- |
|  |  |  | **< 45 years** | | | | | | | | **≥ 45 years** | | | | | | | |
|  |  |  | **rs1050450** | | **rs17650792** | | **rs4880** | | **rs769217** | | **rs1050450** | | **rs17650792** | | **rs4880** | | **rs769217** | |
| **BMD site** | **Model** | **Genotype** | **β (95%CI)** | ***p* value** | **β (95%CI)** | ***p* value** | **β (95%CI)** | ***p* value** | **β (95%CI)** | ***p* value** | **β (95%CI)** | ***p* value** | **β (95%CI)** | ***p* value** | **β (95%CI)** | ***p* value** | **β (95%CI)** | ***p* value** |
| Total hip | Additive |  | -0.007  (-0.033,0.018) | 0.559 | 0.002  (-0.019,0.022) | 0.880 | -0.010  (-0.028,0.009) | 0.308 | -0.016  (-0.035,0.004) | 0.118 | -0.002  (-0.018,0.014) | 0.784 | -0.004  (-0.016,0.009) | 0.588 | 0.012  (0.0006,0.023) | 0.040 | 0.007  (-0.006,0.019) | 0.285 |
|  | Codominant | GG | 0.0 |  | 0.0 |  | 0.0 |  | 0.0 |  | 0.0 |  | 0.0 |  | 0.0 |  | 0.0 |  |
|  |  | GA | 0.010  (-0.019,0.039) | 0.498 | 0.029  (0.002,0.056) | 0.037 | -0.013  (-0.040,0.014) | 0.348 | -0.019  (-0.046,0.007) | 0.154 | -0.005(-0.023,0.014) | 0.632 | 0.0008  (-0.016,0.017) | 0.927 | 0.007(-0.010,0.023) | 0.412 | 0.022  (0.006,0.039) | 0.007 |
|  |  | AA | -0.099  (-0.185,-0.012) | 0.025 | -0.049(-0.102,0.004) | 0.070 | -0.016(-0.057,0.024) | 0.425 | -0.026  (-0.074,0.022) | 0.289 | 0.007(-0.049,0.063) | 0.817 | -0.017(-0.051,0.017) | 0.338 | 0.028(0.003,0.054) | 0.026 | -0.012(-0.042,0.018) | 0.428 |
|  | Dominant | GG | 0.0 |  | 0.0 |  | 0.0 |  | 0.0 |  | 0.0 |  | 0.0 |  | 0.0 |  | 0.0 |  |
|  |  | GA+AA | 0.001  (-0.027,0.030) | 0.925 | 0.017  (-0.009,0.042) | 0.208 | -0.014  (-0.039,0.012) | 0.290 | -0.020  (-0.045,0.005) | 0.112 | -0.004(-0.022,0.014) | 0.692 | -0.002(-0.017,0.014) | 0.843 | 0.011(-0.004,0.027) | 0.152 | 0.017  (0.002,0.033) | 0.031 |
|  | Recesive | GG+GA | 0.0 |  | 0.0 |  | 0.0 |  | 0.0 |  | 0.0 |  | 0.0 |  | 0.0 |  | 0.0 |  |
|  |  | AA | -0.101  (-0.187,-0.015) | 0.021 | -0.059  (-0.112,-0.006) | 0.028 | -0.010(-0.048,0.028) | 0.606 | -0.018  (-0.065,0.029) | 0.455 | 0.008(-0.048,0.063) | 0.790 | -0.017(-0.051,0.017) | 0.321 | 0.025(0.001,0.048) | 0.039 | -0.023  (-0.052,0.007) | 0.127 |
| Femoral neck | Additive |  | -0.013(-0.038,0.012) | 0.301 | -0.002(-0.023,0.019) | 0.831 | -0.013(0.031,0.005) | 0.168 | -0.012(-0.032,0.008) | 0.225 | -0.004(-0.019,0.012) | 0.647 | -0.005(-0.018,0.007) | 0.395 | 0.011(-0.0004,0.022) | 0.058 | 0.008(-0.004,0.020) | 0.171 |
|  | Codominant | GG | 0.0 |  | 0.0 |  | 0.0 |  | 0.0 |  | 0.0 |  | 0.0 |  | 0.0 |  | 0.0 |  |
|  |  | GA | -0.003(-0.032,0.027) | 0.865 | 0.021(-0.006,0.048) | 0.119 | -0.010(-0.037,0.016) | 0.450 | -0.007(-0.033,0.020) | 0.612 | -0.004(-0.022,0.014) | 0.659 | 0.0001(-0.016,0.016) | 0.988 | 0.009(-0.007,0.025) | 0.263 | 0.018(0.003,0.034) | 0.021 |
|  |  | AA | -0.079(-0.166,0.009) | 0.077 | -0.051(-0.105,0.002) | 0.060 | -0.028(-0.068,0.012) | 0.173 | -0.033(-0.081,0.015) | 0.181 | -0.005(-0.059,0.049) | 0.853 | -0.023(-0.056,0.010) | 0.172 | 0.023(-0.001,0.047) | 0.066 | 0.0004(-0.029,0.029) | 0.980 |
|  | Dominant | GG | 0.0 |  | 0.0 |  | 0.0 |  | 0.0 |  | 0.0 |  | 0.0 |  | 0.0 |  | 0.0 |  |
|  |  | GA+AA | -0.008(-0.037,0.020) | 0.555 | 0.010(-0.015,0.036) | 0.432 | -0.014(-0.039,0.011) | 0.269 | -0.011(-0.036,0.014) | 0.385 | -0.004(-0.022,0.013) | 0.641 | -0.003(-0.018,0.012) | 0.699 | 0.012(-0.003,0.027) | 0.119 | 0.016(0.0006,0.031) | 0.041 |
|  | Recesive | GG+GA | 0.0 |  | 0.0 |  | 0.0 |  | 0.0 |  | 0.0 |  | 0.0 |  | 0.0 |  | 0.0 |  |
|  |  | AA | -0.078(-0.165,0.009) | 0.078 | -0.059(-0.112,-0.006) | 0.029 | -0.023(-0.061,0.015) | 0.237 | -0.030(-0.077,0.017) | 0.209 | -0.004(-0.058,0.050) | 0.878 | -0.023(-0.056,0.009) | 0.163 | 0.017(-0.005,0.041) | 0.121 | -0.008(-0.036,0.020) | 0.562 |
| CI= Confidence interval  BMD: Bone Mineral Density  *GPX1*: Glutation Peroxidase 1  *SOD2*: Superoxide Dismutase 2  *CAT*: Catalase  Model adjusted for age, BMI, total energy, smoking status, physical activity, alcohol intake, calcium intake, calcium supplements, THR, vitamin D intake. | | | | | | | | | | | | | | | | | | |

| **Supplementary Table S5.** Association of *GPX1*, *SOD2* and *CAT* variants with low BMD in Mexican women stratified by age. | | | | | | | | | | | | | | | | | | |
| --- | --- | --- | --- | --- | --- | --- | --- | --- | --- | --- | --- | --- | --- | --- | --- | --- | --- | --- |
|  |  |  | **<45 years** | | | | | | | | **≥45 years** | | | | | | | |
|  |  |  | **rs1050450** | | **rs17650792** | | **rs4880** | | **rs769217** | | **rs1050450** | | **rs17650792** | | **rs4880** | | **rs769217** | |
| **BMD site** | **Model** | **Genotype** | **OR**  **(95%CI)** | ***p* value** | **OR**  **(95%CI)** | ***p* value** | **OR**  **(95%CI)** | ***p* value** | **OR**  **(95%CI)** | ***p* value** | **OR (95%CI)** | ***p* value** | **OR (95%CI)** | ***p* value** | **OR**  **(95%CI)** | ***p* value** | **OR**  **(95%CI)** | ***p* value** |
| Total hip | Additive |  | 0.95(0.40-2.22) | 0.898 | 0.90(0.44-1.82) | 0.767 | 1.29(0.73-2.29) | 0.375 | 1.41(0.77-2.61) | 0.268 | 1.02(0.71-1.47) | 0.911 | 1.20(0.90-1.61) | 0.204 | 0.84(0.65-1.10) | 0.205 | 1.01(0.77-1.33) | 0.946 |
|  | Codominant | GG | 1.0 |  | 1.0 |  | 1.0 |  | 1.0 |  | 1.0 |  | 1.0 |  | 1.0 |  | 1.0 |  |
|  |  | GA | 0.73(0.27-1.95) | 0.525 | 0.46(0.18-1.22) | 0.120 | 1.22(0.50-3.01) | 0.660 | 1.44(0.62-3.34) | 0.397 | 1.03(0.68-1.55) | 0.893 | 1.12(0.77-1.61) | 0.557 | 1.17(0.81-1.69) | 0.391 | 0.84(0.58-1.21) | 0.344 |
|  |  | AA | 3.39(0.26-43.94) | 0.351 | 2.32(0.54-10.09) | 0.260 | 1.72(0.53-5.63) | 0.367 | 1.96(0.47-8.12) | 0.356 | 0.99(0.23-4.22) | 0.993 | 1.73(0.81-3.70) | 0.160 | 0.49(0.26-0.92) | 0.026 | 1.33(0.70-2.52) | 0.382 |
|  | Dominant | GG | 1.0 |  | 1.0 |  | 1.0 |  | 1.0 |  | 1.0 |  | 1.0 |  | 1.0 |  | 1.0 |  |
|  |  | GA+AA | 0.82(0.32-2.10) | 0.675 | 0.63(0.27-1.51) | 0.301 | 1.34(0.58-3.09) | 0.486 | 1.51(0.68-3.38) | 0.311 | 1.03(0.69-1.53) | 0.899 | 1.18(0.83-1.68) | 0.350 | 1.00(0.71-1.42) | 1.000 | 0.90(0.64-1.28) | 0.572 |
|  | Recesive | GG+GA | 1.0 |  | 1.0 |  | 1.0 |  | 1.0 |  | 1.0 |  | 1.0 |  | 1.0 |  | 1.0 |  |
|  |  | AA | 3.70(0.29-47.56) | 0.316 | 2.93(0.69-12.37) | 0.143 | 1.56(0.53-4.66) | 0.422 | 1.66(0.42-6.48) | 0.467 | 0.99(0.23-4.18) | 0.987 | 1.68(0.78-3.51) | 0.187 | 0.45(0.25-0.82) | 0.009 | 1.45(0.78-2.67) | 0.239 |
| Femoral neck | Additive |  | 1.63(0.83-3.20) | 0.153 | 1.37(0.80-2.37) | 0.253 | 1.44(0.88-2.35) | 0.149 | 1.48(0.88-2.47) | 0.138 | 1.36(0.97-1.90) | 0.075 | 1.36(1.03-1.79) | 0.028 | 0.81(0.63-1.03) | 0.087 | 0.83(0.64-1.07) | 0.148 |
|  | Codominant | GG | 1.0 |  | 1.0 |  | 1.0 |  | 1.0 |  | 1.0 |  | 1.0 |  | 1.0 |  | 1.0 |  |
|  |  | GA | 1.27(0.58-2.79) | 0.545 | 0.76(0.35-1.65) | 0.494 | 1.60(0.75-3.39) | 0.224 | 1.43(0.70-2.92) | 0.321 | 1.43(0.96-2.11) | 0.078 | 1.21(0.86-1.71) | 0.269 | 0.72(0.50-1.01) | 0.059 | 0.78(0.55-1.10) | 0.150 |
|  |  | AA | 8.71(0.99-76.04) | 0.050 | 4.07(1.23-13.48) | 0.021 | 1.94(0.68-5.54) | 0.216 | 2.26(0.70-7.34) | 0.174 | 1.48(0.47-4.66) | 0.503 | 2.49(1.15-5.39) | 0.021 | 0.73(0.43-1.24) | 0.247 | 0.75(0.41-1.39) | 0.359 |
|  | Dominant | GG | 1.0 |  | 1.0 |  | 1.0 |  | 1.0 |  | 1.0 |  | 1.0 |  | 1.0 |  | 1.0 |  |
|  |  | GA+AA | 1.46(0.69-3.10) | 0.319 | 1.06(0.53-2.12) | 0.868 | 1.67(0.82-3.40) | 0.156 | 1.55(0.79-3.04) | 0.203 | 1.43(0.98-2.09) | 0.066 | 1.33(0.95-1.85) | 0.093 | 0.72(0.52-0.99) | 0.049 | 0.77(0.55-1.07) | 0.124 |
|  | Recesive | GG+GA | 1.0 |  | 1.0 |  | 1.0 |  | 1.0 |  | 1.0 |  | 1.0 |  | 1.0 |  | 1.0 |  |
|  |  | AA | 8.13(0.95-69.84) | 0.056 | 4.45(1.38-14.35) | 0.012 | 1.52(0.58-3.99) | 0.390 | 1.92(0.62-5.92) | 0.255 | 1.37(0.44-4.29) | 0.592 | 2.32(1.08-4.98) | 0.030 | 0.87(0.53-1.43) | 0.575 | 0.84(0.47-1.53) | 0.576 |
| Lumbar spine | Additive |  | 1.04(0.59-1.85) | 0.883 | 1.17(0.74-1.84) | 0.506 | 1.26(0.84-1.89) | 0.256 | 0.88(0.57-1.38) | 0.586 | 0.95(0.69-1.30) | 0.759 | 0.99(0.76-1.27) | 0.914 | 0.86(0.69-1.08) | 0.194 | 0.79(0.62-1.01) | 0.056 |
|  | Codominant | GG | 1.0 |  | 1.0 |  | 1.0 |  | 1.0 |  | 1.0 |  | 1.0 |  | 1.0 |  | 1.0 |  |
|  |  | GA | 0.87(0.45-1.68) | 0.675 | 0.92(0.50-1.69) | 0.789 | 1.64(0.89-3.01) | 0.111 | 1.06(0.60-1.87) | 0.851 | 0.96(0.66-1.39) | 0.828 | 0.98(0.71-1.36) | 0.905 | 0.91(0.65-1.26) | 0.565 | 0.71(0.51-0.98) | 0.035 |
|  |  | AA | 2.77(0.45-16.89) | 0.270 | 2.06(0.69-6.20) | 0.197 | 1.33(0.54-3.28) | 0.541 | 0.52(0.14-1.90) | 0.321 | 0.88(0.30-2.54) | 0.806 | 0.98(0.49-1.97) | 0.965 | 0.71(0.44-1.16) | 0.171 | 0.74(0.41-1.33) | 0.312 |
|  | Dominant | GG | 1.0 |  | 1.0 |  | 1.0 |  | 1.0 |  | 1.0 |  | 1.0 |  | 1.0 |  | 1.0 |  |
|  |  | GA+AA | 0.95(0.50-1.78) | 0.865 | 1.05(0.59-1.85) | 0.866 | 1.56(0.88-2.79) | 0.129 | 0.96(0.56-1.67) | 0.897 | 0.95(0.66-1.36) | 0.787 | 0.98(0.72-1.34) | 0.904 | 0.86(0.63-1.17) | 0.345 | 0.71(0.52-0.97) | 0.031 |
|  | Recesive | GG+GA | 1.0 |  | 1.0 |  | 1.0 |  | 1.0 |  | 1.0 |  | 1.0 |  | 1.0 |  | 1.0 |  |
|  |  | AA | 2.88(0.48-17.42) | 0.250 | 2.12(0.72-6.24) | 0.172 | 1.03(0.44-2.38) | 0.948 | 0.51(0.14-1.80) | 0.294 | 0.88(0.31-2.55) | 0.818 | 0.99(0.50-1.97) | 0.981 | 0.75(0.47-1.18) | 0.211 | 0.87(0.49-1.54) | 0.630 |
| CI= Confidence interval  *GPX1*: Glutation Peroxidase 1  *SOD2*: Superoxide Dismutase 2  *CAT*: Catalase  Model adjusted for age, BMI, total energy, smoking status, physical activity, alcohol intake, calcium intake, calcium supplements, THR, vitamin D intake. | | | | | | | | | | | | | | | | | | |

| **Supplementary Table S6.** Demographic characteristics of the study population by rs769217 (*CAT*) and rs4880 (*SOD2*). | | | | | | | |
| --- | --- | --- | --- | --- | --- | --- | --- |
|  | **rs769217** | | | **rs4880** | | | |
|  | CC | CT+TT |  | GG | GA | AA |  |
| **Variable** | n=631 | n=623 | *p* value | n=535 | n=579 | n=146 | *p* value |
| Age (yr.) | 52(41-61) | 54(43-64) | 0.0252 | 53(42-63) | 53(42-63) | 54(44-62) | 0.3300 |
| BMI^a^ (kg/m^2^) | 26.8(23.8-30.1) | 26.7(24.1-29.9) | 0.9644 | 26.9(24.2-30.1) | 26.3(23.7-30) | 27.3(23.7-29.8) | 0.4312 |
| Glucose (mg/dL) | 95(89-104) | 96(90-106) | 0.0298 | 96(90-105) | 96(90-104) | 95(90-103) | 0.2816 |
| IFG^b^ (%) | 5.5 | 5.1 | 0.7417 | 5.6 | 5.6 | 4.8 | 0.703 |
| Diabetes (%) | 14.3 | 18.6 | 0.0321 | 18.1 | 14.9 | 16.4 | 0.6306 |
| Total cholesterol (mg/dL) | 197(170-223) | 199(174-231) | 0.0452 | 196(170-224) | 201(174-229) | 197(170-222) | 0.4305 |
| High cholesterol (%) | 47.4 | 49.9 | 0.3758 | 45.6 | 60 | 47.3 | 0.7123 |
| Energy (kcal/día) | 1695(1288-2238) | 1705(1271-2225) | 0.9104 | 1674(1255-2188) | 1733(1328-2275) | 1688(1317-2174) | 0.442 |
| BMD^c^ total hip (g/cm^2^) | 0.970(0.877-1.074) | 0.966(0.871-1.065) | 0.6868 | 0.962(0.876-1.076) | 0.971(0.867-1.062) | 0.969(0.896-1.059) | 0.3022 |
| BMD^c^ femoral neck (g/cm^2^) | 0.937(0.835-1.029) | 0.930(0.833-1.025) | 0.6608 | 0.927(0.830-1.029) | 0.934(0.830-1.028) | 0.934(0.849-1.015) | 0.4976 |
| BMD^c^ lumbar spine (g/cm^2^) | 1.060(0.956-1.170) | 1.060(0.952-1.183) | 0.6482 | 1.073(0.950-1.172) | 1.066(0.960-1.173) | 1.090(0.954-1.183) | 0.2376 |
| Retinol (UI/day) | 7225(6799-7839) | 7233(6767-7796) | 0.9207 | 7233(6799-7762) | 7266(6788-7927) | 7083(6704-7603) | 0.0237 |
| Vitamin C (mg/day) | 311(230-389) | 305(232-389) | 0.5746 | 312(232-382) | 310(231-394) | 288(213-369) | 0.0884 |
| Vitamin E (µg/day) | 6.8(5.8-8.0) | 6.8(5.7-8.0) | 0.8155 | 6.8(5.9-8.0) | 6.8(5.7-8.1) | 6.4(5.5-7.4) | 0.0241 |
| Selenium (µg/day) | 39.7(31.3-49.6) | 39.0(29.0-48.7) | 0.2824 | 39.9(31-49.7) | 38.4(30.2-49.2) | 39.2(30.3-47.5) | 0.2263 |
| Zinc, (mg/day) | 6.5(5.7-7.5) | 6.5(5.8-7.5) | 0.9029 | 6.5(5.8-7.4) | 6.5(5.7-7.5) | 6.7(5.8-7.6) | 0.1017 |
| Magnesium (mg/day) | 329(293-370) | 328(293-376) | 0.4163 | 328(291-376) | 330(295-369) | 325(288-373) | 0.2918 |
| 0-4^d^ (%) | 55.9 | 57.1 | 0.6682 | 57.8 | 55.8 | 53.4 | 0.3414 |
| 5-6 ^d^ (%) | 44.1 | 42.6 | 0.7107 | 42.2 | 42.2 | 46.6 | 0.5008 |
| Folate^d^ (µg/day) | 319.6(267.2-389.8) | 313.3(261-388.5) | 0.1872 | 314.2(267.6-381.4) | 320.7(262.2-394.2) | 313.0(253.7-378.0) | 0.2123 |
| Omega 6^d^ (g/day) | 11.6(9.6-13.3) | 11.3(9.45-13.1) | 0.2451 | 11.6(9.7-13.3) | 11.3(9.4-13.2) | 11.4(9.6-13.2) | 0.3942 |
| Saturated fat ^d^ (g/day) | 15.0(11.9-17.9) | 14.6(11.9-17.6) | 0.3301 | 15.1(12.0-17.9) | 14.6(11.8-17.4) | 15.4(12.5-18.2) | 0.1679 |
| Fiber ^d^ (g/day) | 26.8(21.5-32.4) | 26.7(22.7-32.2) | 0.5490 | 26.9(22.0-32.9) | 26.7(22.8-32.0) | 25.1(20.0-31.0) | 0.0184 |
| Riboflavin^d^ (mg/day) | 1.53(1.30-1.84) | 1.50(1.26-1.77) | 0.1438 | 1.50(1.28-1.77) | 1.52(1.27-1.81) | 1.58(1.36-1.93) | 0.008 |
| Vitamin B6^d^ (mg/day) | 1.65(1.41-1.96) | 1.64(1.40-1.88) | 0.2071 | 1.65(1.43-1.93) | 1.65(1.41-1.94) | 1.60(1.35-1.84) | 0.0736 |
| Vitmina D^d^ (UI/day) | 4.3(2.9-6.1) | 4.1(2.6-6.0) | 0.1849 | 4.1(2.6-5.8) | 4.0(2.7-6.0) | 4.8(3.5-6.9) | 0.0004 |
| Median (P25-P75)  ^a^BMI = Body Mass Index  ^b^IFG = Impaired Fasting Glucose  ^c^BMD = Bone Mineral Density  ^d^DAQs = Dietary antioxidant quality score | | | | | | | |
